# The Association between Dental Pain and Psychological Symptoms: Evidence from a Population-Based Study in Indonesia

**DOI:** 10.1055/s-0043-1774320

**Published:** 2023-11-23

**Authors:** Ninuk Hariyani, Tantry Maulina, Rahul Nair

**Affiliations:** 1 Department of Dental Public Health, Faculty of Dental Medicine, Universitas Airlangga, Surabaya, Indonesia; 2 Australian Research Centre for Population Oral Health, Adelaide Dental School, The University of Adelaide, Adelaide, South Australia, Australia; 3 Department of Oral and Maxillofacial Surgery , Faculty of Dentistry, Universitas Padjadjaran, Bandung, Indonesia; 4 Biomedical Science Program, Radboud University, Nijmegen, The Netherlands; 5 Department of Dentistry - Quality and Safety of Oral Healthcare, Radboud University Medical Center, Radboud Institute for Health Sciences, Nijmegen, The Netherlands

**Keywords:** dental pain, depression, stress, Indonesia, population-based study, epidemiology

## Abstract

**Objectives** This study aimed to determine the prevalence and distribution of dental pain and its association with psychological symptoms: stress and depression.

**Materials and Methods** The 2014 Indonesia Family Life Survey data were used for the abovementioned purposes. Records of self-reported dental pain and stress as well as the occurrence of depression based on the 10-item Center for Epidemiologic Studies-Depression scale were analyzed. Multivariable ordinal regression models were fitted to test the hypothesized associations between dental pain and each of the psychological symptoms, controlling for age, sex, education, wealth, and religiosity.

**Results** Dental pain prevalence among Indonesian population was approximately 15%. The adjusted odds ratios (ORs) from the ordinal regression models indicated that dental pain was related to the increase of the stress and depression level (OR: 1.31, 95% confidence interval [CI]: 1.14–1.49 and OR: 1.59, 95% CI: 1.41–1.79, respectively). Higher education, higher wealth, and being male are risk factors for stress. However, higher wealth and being male are protective factors for depression. Furthermore, higher religious level was a protective factor for both stress and depression.

**Conclusion** Dental pain was associated with a higher level of stress and a higher score of depression, even after being adjusted with age, sex, education, wealth, and religiosity.

## Introduction

One of the most experienced orofacial pains is those of the odontogenic origin, which is known as tooth pain or dental pain. 1 2 3 Dental pain can be defined as pain that originated from the teeth or the supporting tissues as a consequence of an occurring disease or injury of the teeth. 2 Studies on the prevalence or incidence of dental pain varies. A study about the prevalence of dental pain on Mexican population aged between 16 and 25 years old revealed that dental pain occurred in 34% of the total participants. 4 Another study conducted by Horst et al about the prevalence of orofacial pain in the United States showed that at least 9.1% of the participants ( *n* = 1,668) have had dental pain for the last year. In Indonesia, a study that was conducted on 5,412 participants aged 18 years old and older revealed that 2,892 of the participants experienced dental pain in the last 6 months. 5

Aside from its high prevalence, dental pain is also known for its impacts on the sufferer's (oral health) quality of life. 2 6 7 In a study conducted by Samuel et al about the impact of dental pain on the patient's oral health quality of life (OHQoL), it was reported that higher self-reported pain score and longer pain duration negatively impact the OHQoL. 7 Additionally, Svensson et al who conducted research about the impact of dental pain on OHQoL also reported that lower OHQoL was shown by subjects who reported dental pain, and that dental pain was associated with the probability of poor OHQoL. 2 Considering the nature of pain in general that has been associated with several psychological symptoms, the association between orofacial pain, including dental pain, and several psychological symptoms are also being explored. 7 8 9

In a preliminary study conducted by Shafira et al 10 on patients with acute orofacial pain due to oral and maxillofacial infection or oral and maxillofacial trauma, it was shown that the level of orofacial pain was significantly related to jaw and facial activities limitation ( *p* < 0.01) and that the limitation of the jaw and facial activities was significantly related ( *p* = 0.001) to stress. Interestingly, no significant association was found between stress and acute orofacial pain. 10 In regard to depression, based on this preliminary result, it would be of importance to investigate how dental pain, being the most experienced type of orofacial pain, might correlate with stress. Yang et al has conducted a study about the association between depression and dental pain and showed that an increase in self-reported dental pain was shown in patients with depression. It was also concluded that dental pain was found to be associated with depression. 11

Considering the lack of evidence regarding the association between dental pain and two most common psychological symptoms, namely, stress and depression, provided by previous studies, further study analyzing the association between these variables is of importance. Besides reporting the prevalence and distribution of dental pain among Indonesian adults, the current study aimed on evaluating the hypothesized positive association between dental pain and two psychological symptoms, which are stress and depression.

## Materials and Methods

### Data and Variables

The secondary data analysis used data from the 2014 Indonesia Family Life Survey (IFLS wave 5), available from RAND at http://www.rand.org/labor/FLS/IFLS.html . The IFLS is an ongoing multipurpose survey already conducted in 5 waves, with data collected in 1993, 1997, 2000, 2007, and 2014. The survey was started in collaboration with RAND Corporation (United States) and collected a representative data of approximately 83% of the entire Indonesian population. Stratified sampling was conducted by province. Furthermore, households to be interviewed are randomly selected within the provinces to include both rural and urban areas. The detail of the survey procedure can be found elsewhere. 12 13

All variables including the exposure (dental pain), the outcome of interest (psychological symptoms—stress and depression), and the covariates (age, sex, educational level, wealth, and religiosity) were gathered from the ILFS 2014 questionnaire. The latest survey was selected as it reflected the latest conditions and contained the largest sample size among all the waves. The covariates included in the analysis were selected before the analysis based on the previous literatures identifying possible associations. 14 15 16 17 18 Previous research has highlighted the association of education, wealth, and religiosity with psychological symptoms, both stress and depression. Moreover, dental pain, stress, and depression also varied due to age and sex.

Dental pain was reported by the respondents when they were asked about experiencing a toothache in the last 4 weeks. The possible answer is yes versus no. The level of stress was self-reported on a global item measuring stress during work by the respondents as having no stress (based on answer “None/Almost none of the time”), low stress (based on answer “Some of the time”), or high stress (based on answer “Most of the time” or “Almost all the time”). Depression was measured using the 10-item Center for Epidemiologic Studies-Depression (CES-D) scale. 19 This scale was developed to measure depressive symptoms in the general population. 20 It has been proven to be valid in various Asian settings, including in Indonesia. 21 In total, there are 10 depressive symptoms listed on the CES-D scale and each respondent need to report how often in the past week they had experienced them. The responses were recorded using a four-category rating scale that ranged from 0 to 3 (0 = rarely or none of the time or 0–1 day in a week; 1 = some or little of the time or 1–2 days in a week; 2 = moderately or much of the time or 3–4 days in a week; 3 = most or almost all the time or 5–7 days in a week). After reverse-coding the positively phrased items, we calculated the scores as the sum of these responses (theoretical range 0–30, where 0 represents people with no depression, and 1–30, ascending levels of depression). 22 The CES-D commonly has a positive or right-skewed distribution 20 ; thus, groups with higher means also tend to have higher variances. Therefore, 3 categories were created to assess the effect of various cutoffs on the relationship between the depression and dental pain. Uncertainty regarding the appropriate cutoffs in this population lead to the creation of 3 categories in this study, following previous research. 23 Thus, the CES-D score was categorized into low, intermediate, and high symptom of depression (with corresponding CES-D score of 0–5, 6–10, and > 10, respectively).

The covariates included the standard confounding factors namely, age and sex. In this research, age was treated as a continuous covariate, while sex was entered as a dummy variable (male and female). The other covariates include education, wealth, and religiosity. Education was the highest formal educational level, self-reported by the participants. Participants' wealth was grouped (less advantaged: first and second quintile; more advantaged: third–fifth quintile) according to household assets and household characteristics 24 using principal component analysis. 25 Household assets included house and land occupied by the household, other house/building, land (not used for house or farm), vehicles (cars, boats, bicycles, motorbikes), household appliances (radio, tape recorder, television, fridge, sewing or washing machine, video compact disc player, mobile phone, and others), savings/certificate of deposits/stocks, jewelry, as well as household furniture and utensils. Household characteristics included access to pipe water both for drinking and other household needs, access to toilet, access to electricity, and cooking with electric/gas stove. Religiosity was measured based on a single question of “how religious are you?,” in which we recoded the answer of very religious and religious into religious group and the answer of somewhat religious and not religious into nonreligious group.

### Statistical Analyses

The statistical analyses were performed in SAS-callable (Research Triangle Institute, North Carolina, United States). Descriptive characteristics of the study participants were estimated. Bivariate analyses were performed using chi-squared test for the categorical exposure of interest and the categorical covariate and correlation for the continuous covariate for each of the psychological symptoms—stress and depression. Finally, multivariable ordinal regressions were used to estimate the odds ratios (ORs) of experiencing psychological symptoms and their 95% confidence intervals (95% CI). Three models were developed sequentially in each psychological symptoms—stress and depression. The first model was the null model showing the unadjusted association of dental pain and the psychological symptoms. The second model was the basic model, adjusted with age and sex, while the third model was the fully adjusted model.

### Ethical Approval

Ethical approval for IFLS 2014 was obtained from Gajah Mada University in Indonesia and the Institutional Review Board at RAND Corporation in the United States. This study involved only a secondary analysis; thus, no new ethic clearance was required.

## Results

All samples with full value of all variables included were used in the analysis ( *N* = 7,375). Table 1 shows the characteristics of the study participants. About 15% of the study participant experienced dental pain in the previous months. Approximately 9% (95% CI = 8.38–9.68) and 18.5% (95% CI =17.60–19.37) of the respondents reported high level of stress and high symptoms of depression, respectively. The mean age of the respondents was 37.7 years old and around 24% of them were male. Respondents with education less than a secondary school was around 56.5%, while respondents living in less advantaged wealth and not religious were around 41 and 20%, respectively.

**Table 1:** Characteristics of the study participants

Characteristics	All analyzed participants
	%[CI] or mean [CI]
	***N*** ** = 7,375**
Dental pain	
Yes	15.17 [14.35–15.99]
No	84.83 [84.01–85.65]
Level of stress	
No stress	63.95 [62.85–65.04]
Low stress	27.02 [26.01–28.04]
High stress	9.03 [8.38–9.68]
Depression (ordinal scale based on CES-D score)	
Low (0–5)	51.89 [50.75–53.03]
Mild (6–10)	29.63 [28.58–30.67]
Severe (> 10)	18.48 [17.60–19.37]
Age	37.77 [37.51–38.02]
Sex	
Male	23.63 [22.66–24.60]
Female	76.37 [75.40–77.34]
Education	
Secondary school or less	56.46 [55.33–57.59]
High school	28.16 [27.14–29.19]
College or more	15.38 [14.55–16.20]
Wealth	
Less advantaged	40.79 [39.66–41.91]
More advantaged	59.21 [58.09–60.34]
Religiosity	
Not religious	20.15 [19.23–21.06]
Religious	79.85 [78.94–80.77]

Abbreviations: CES-D, Center for Epidemiologic Studies-Depression; CI, confidence interval.

Table 2 reveals the association of dental pain and other covariates with psychological symptoms (stress and depression). Person with dental pain showed higher stress and depression than person without dental pain. Age has negative association with all psychological symptoms (stress and depression). Women showed lower stress but higher depression than men. People with higher education and higher wealth showed higher work stress level. On the other hand, people with higher education and higher wealth showed lower depression symptoms. Person with higher religious level showed lower stress and depression symptoms.

**Table 2:** Bivariate analyses (unadjusted analysis) of the associations of dental pain with psychological symptoms (stress and depression)

Characteristics	Association with stress			Significance	Association with depression			Significance
	No stress	Low stress	High stress		Low depression	Mild depression	Severe depression	
	%	%	%		%	%	%	
*N* = 7,375								
Dental pain								
No	**64.4**	**27.0**	**8.6**	**0.01**	**53.6**	**29.0**	**17.3**	**< 0.001**
Yes	**61.6**	**27.1**	**11.3**		**42.2**	**33.0**	**24.8**	
Age (correlation coefficient)	**–0.13**			**< 0.001**	** –0.08**			**< 0.001**
Gender								
Male	**55.8**	**30.7**	**13.5**	**< 0.001**	**54.0**	**29.0**	**17.0**	**0.09**
Female	**66.5**	**25.9**	**7.7**		**51.2**	**29.8**	**18.9**	
Education								
Secondary school or less	**75.1**	**18.3**	**6.6**	**< 0.001**	**51.7**	**28.7**	**19.6**	**< 0.001**
High school	**56.0**	**32.9**	**11.0**		**51.1**	**30.1**	**18.8**	
College or more	**37.3**	**48.3**	**14.4**		**53.9**	**32.3**	**13.8**	
Wealth								
Less advantaged	**73.4**	**19.6**	**7.0**	**< 0.001**	**49.8**	**29.6**	**20.6**	**< 0.001**
More advantaged	**57.4**	**32.2**	**10.4**		**53.3**	**29.7**	**17.0**	
Religiosity								
Not religious	**56.2**	**32.2**	**11.6**	**< 0.001**	**43.8**	**34.8**	**21.4**	**< 0.001**
Religious	**65.9**	**25.7**	**8.4**		**53.9**	**28.3**	**17.7**	

Note: Bold = significant in unadjusted analysis. Tests were performed using chi-squared test and correlation.

Tables 3 and 4 show multivariable analysis of the association of dental pain with psychological symptoms (depression and stress, respectively) in three models, namely, the null model, basic model, and fully adjusted model. Dental pain was associated with the increase of stress and depression in all models. The adjusted ORs from the fully adjusted ordinal regression models indicated that dental pain was related to 1.31 increase of stress level (OR: 1.31, 95% CI: 1.14–1.49), while people having dental pain was related to 1.59 increase of depression level (OR: 1.59, 95% CI: 1.41–1.79). Age had a negative association with all psychological symptoms (OR: 0.99, 95% CI: 0.98–0.99 for both stress and depression). Being male (OR: 1.57, 95% CI: 1.40–1.75), having higher education (OR: 3.73, 95% CI: 3.25–4.28), and having higher wealth (OR: 1.45, 95% CI: 1.30–1.62) were risk factors for stress. However, being male (OR: 0.85, 95% CI: 0.76–0.94) and having higher wealth (OR: 0.85, 95% CI: 0.77–0.93) were protective factors for depression. Furthermore, higher religious level were protective factors for both stress and depression (OR: 0.74, 95% CI: 0.66–0.83 and OR: 0.74, 95% CI: 0.67–0.83, respectively).

**Table 3:** Multivariable analysis of the association of dental pain with stress level

	Null model of stress a OR [CI]	Stress	Fully adjusted model of stress a OR [CI]
		** Basic model of stress a** **OR [CI]**	
Dental pain			
No (ref)	−	−	−
Yes	**1.16 [1.02–1.32]**	**1.20 [1.06–1.37]**	**1.31 [1.14–1.49]**
Age		**0.98 [0.97–0.98]**	**0.99 [0.98–0.99]**
Gender			
Male (ref)		**1.58 [1.42–1.76]**	**1.57 [1.40–1.75]**
Female		**-**	**-**
Education			
Secondary school or less (ref)			**-**
High school			**1.86 [1.66–2.09]**
College or more			**3.73 [3.25–4.28]**
Wealth			
Less advantaged (ref)			**-**
More advantaged			**1.45 [1.30–1.62]**
Religiosity			
Not religious (ref)			**0.74 [0.66–0.83]**
Religious			

Abbreviations: CI, confidence interval; OR, odd ratio.

Note: Bold = significant.

a Converged model using ordinal regression.

**Table 4:** Multivariable analysis of the association of dental pain with depression symptoms

	Null model of depression a OR [CI]	Depression	Fully adjusted model of depression a OR [CI]
		** Basic model of depression a** **OR [CI]**	
Dental pain			
No (ref)			
Yes	**1.58 [1.41–1.78]**	**1.61 [1.43–1.81]**	**1.59 [1.41–1.79]**
Age		**0.99 [0.98–0.99]**	**0.99 [0.98–0.99]**
Gender			
Male (ref)		**0.89 [0.80–0.98]**	**0.85 [0.76–0.94]**
Female		**-**	**-**
Education			
Secondary school or less (ref)			**-**
High school			0.96 [0.86–1.07]
College or more			0.88 [0.77–1.00]
Wealth			
Less advantaged (ref)			**-**
More advantaged			**0.85 [0.77–0.93]**
Religiosity			
Not religious (ref)			**-**
Religious			**0.74 [0.67–0.83]**

Abbreviations: CI, confidence interval; OR, odd ratio.

Note: Bold = significant.

a Converged model using ordinal regression.

## Discussion

The current study revealed that all participants claimed to experience different levels of depression, while stress was only experienced by 36.05% participants ( Table 1 ). This result is in line with previous study by Salari et al, about the prevalence of several psychological symptoms, namely, anxiety, stress, and depression, where the prevalence of depression was found to be the highest among the three symptoms. 26 A similar result was also shown by a study conducted by Parvar et al regarding the prevalence of several psychological conditions. 27 Dental pain was found in 15% of the participants, suggesting a high number considering the pain experience must be something the participant experienced or have been experiencing in the last 4 weeks. This high dental pain prevalence was also shown by previous studies. 2 28

When tested for correlation, significant associations between dental pain and stress and depression ( Table 2 ) were found. As there have been very few studies that correlates dental pain and stress, the possible explanation that might underline the phenomenon is suspected to be similar with the one that underlies the correlation between stress and oral and facial pain in general, considering that all pain that occur within the orofacial area is transmitted through the trigeminal system to the brain in the same manner. As experiencing acute dental pain is suspected to be a stressful event for the patient, the stress is expected to activate the sympathoadrenal axis to secrete catecholamines and the hypothalamic-pituitary-adrenal axis to secrete cortisol. 29 As cortisol has been known for its effect on inflammation and the activation of the sympathetic nervous system will also affect inflammation, 29 30 including inflammation of the tooth 31 that can lead to dental pain, 32 the situation will lead to more stressful situation for the patient, creating a repeated cycle of events. Which of course, reflects the association between stress and dental pain that can be used to explain the results of the current study.

Concerning the association between pain and depression, several studies have documented this association. 33 34 35 In a study that evaluated long-term depression, greater pain severity at baseline, a greater number of pain locations, and longer pain duration significantly increased the risk of still experiencing depression after 2 years' period. 36 37 Interestingly, results from previous studies revealed that the brain areas that are affected in depressed patients, namely, the prefrontal cortex, anterior cingulate cortex, nucleus accumbens, hippocampus, and the amygdala, are also the areas that are found to be affected in acute and chronic pain patients. 37 In regard to hippocampus and amygdala, these brain structures have been identified for their role in pain-induced depressive behaviors. In an inflammatory pain model on rodents, neurometabolic changes that can lead to a decreased hippocampus serotonin level have been identified as the causing factor of depressive symptoms. 37 38

Concerning the result of the current study about the association between depression and dental pain, in our study, it was revealed that dental pain is a risk factor for both stress and depression. These particular results are in the opposite direction of previous studies where the psychological symptoms were the risk factors for dental pain, which might be explained by the suspected bidirectional relationship between pain and psychological variables based in previous studies. 39 40 In a study conducted by Haug and Marthinussen, it was revealed that patient with acute dental pain showed higher level of several stress biomarkers, indicating the association between acute dental pain and stress. 29 A study by Yang et al about the relationship between dental pain and QoL and mental health of the South Korean adults, showed that patients with depression showed higher prevalence of dental pain. 41 In another study conducted by Park et al about depression and its association with oral health behavior, it was also revealed that patients with depression reported more frequent occurrences of dental pain. 42

Another finding of the current study is the negative association between age and all psychological variables. This result is in line with a meta-analysis on 192 epidemiological studies conducted by Solmi et al, that revealed the peak age of any mental disorders is 14.5 years of age. 43 In another study conducted by Fields et al, it was also revealed that older age is associated with lower stress and depressive symptomatology. 44 Additionally, this current study also found that women were more likely to have depression compared with men, which is in line with the findings of Villaroel and Terlizzi. 45 The next finding in this current study is that people with higher education and higher wealth showed higher work stress level and that people with higher education and higher wealth showed lower depression symptoms. This is an interesting finding as previous studies actually showed that higher work stress is usually shown by people with lower educational level as well as low financial status. 46 47 These differences of course spark the importance of further research.

The correlation analysis also showed that person with higher religious level showed lower stress and depression symptoms. This finding supports the findings of a previous study conducted by Mahamid and Bdier concerning the association between positive religious coping with perceived stress and depressive symptoms where negative correlations were found for positive religious coping and perceived stress as well as positive religious coping and depressive symptoms. It is hypothesized that when a person is practicing positive religious coping, they begin to perceive strength in their relationship with God, leading toward a positive mind when dealing with a stressful event, diminishing the harmful impact of depressive symptoms. 48

The last part of the analysis performed in this study confirmed the previous correlation analysis. The analysis showed that people with dental pain have an increased possibility of experiencing stress and depression compared with those without dental pain. A more similar result is also shown by a study conducted by Yang et al, where patients with dental pain have increased OR of having lower mental health status compared with those who did not have dental pain. 41 It was also found that being male and having higher wealth status provided a protective effect toward depression, which is in line with a literature study performed by Mofatteh about the risk factors of stress, anxiety, and depression. 49 Lastly, it was found that higher religious level was a protective factor for stress and depression. This finding is supported by previous study conducted by Ronneberg et al, that showed how religiosity provide a protective effect for depression. 50

The use of the IFLS data, which is representative of approximately 83% of the entire Indonesian population, was the strength of this study. The study is also among the few reporting the relationship between psychological factors and dental pain with a representative sample. However, the data used in this current analysis was the cross-sectional data of the wave 5 of IFLS, creating a limitation of the study. The cross-sectional nature of the data does not establish temporal ordering between stress, depression, and dental pain. Further analysis using the longitudinal part of the IFLS data could be used to conform the finding. Furthermore, self-reported stress using a single global question could be a limitation as it may not provide as rich stress description as that measured using a validated questionnaire such as the perceived stress scale. However, self-reported stress using a single global question also has been found to be valid and reliable in the previous research. 51

## Conclusion

Based on the primary analysis, our findings suggest that dental pain was associated with a higher level of stress and a higher score of depression. Additionally, our secondary analysis suggested that there are several risk factors for stress, namely, being male and having higher educational attainment and wealth. As for depression, our secondary analysis suggested that being male and having higher wealth status provided a protective effect toward depression. Furthermore, higher religious level was a protective factor for both stress and depression.
